# Mining for Candidate Genes in an Introgression Line by Using RNA Sequencing: The Anthocyanin Overaccumulation Phenotype in *Brassica*

**DOI:** 10.3389/fpls.2016.01245

**Published:** 2016-08-22

**Authors:** Lulu Xie, Fei Li, Shifan Zhang, Hui Zhang, Wei Qian, Peirong Li, Shujiang Zhang, Rifei Sun

**Affiliations:** Department of Chinese Cabbage, Institute of Vegetables and Flowers, Chinese Academy of Agricultural SciencesBeijing, China

**Keywords:** transcriptome analysis, RNA-seq, introgression lines, *Brassica*, anthocyanin

## Abstract

Introgression breeding is a widely used method for the genetic improvement of crop plants; however, the mechanism underlying candidate gene flow patterns during hybridization is poorly understood. In this study, we used a powerful pipeline to investigate a Chinese cabbage (*Brassica rapa* L. ssp. *pekinensis*) introgression line with the anthocyanin overaccumulation phenotype. Our purpose was to analyze the gene flow patterns during hybridization and elucidate the genetic factors responsible for the accumulation of this important pigment compound. We performed RNA-seq analysis by using two pipelines, one with and one without a reference sequence, to obtain transcriptome data. We identified 930 significantly differentially expressed genes (DEGs) between the purple-leaf introgression line and *B. rapa* green cultivar, namely, 389 up-regulated and 541 down-regulated DEGs that mapped to the *B. rapa* reference genome. Since only one anthocyanin pathway regulatory gene was identified, i.e., *Bra037887* (*bHLH*), we mined unmapped reads, revealing 2031 *de novo* assembled unigenes, including *c3563g1i2*. Phylogenetic analysis suggested that *c3563g1i2*, which was transferred from the *Brassica* B genome of the donor parental line *Brassica juncea*, may represent an R2R3-MYB transcription factor that participates in the ternary transcriptional activation complex responsible for the anthocyanin overaccumulation phenotype of the *B. rapa* introgression line. We also identified genes involved in cold and light reaction pathways that were highly upregulated in the introgression line, as confirmed using quantitative real-time PCR analysis. The results of this study shed light on the mechanisms underlying the purple leaf trait in *Brassica* plants and may facilitate the use of introgressive hybridization for many traits of interest.

## Introduction

Despite advances in genetic engineering, introgression breeding is still a widely used technique for genetically improving crop plants. Introgression breeding begins with interspecific hybridization between a recipient and a donor plant, followed by backcrosses to the recipient plant. After selection for the trait of interest, recipient crop introgression lines will have chromosome segments containing the target genes (Twyford and Ennos, 2012). Genetic materials transferred via this process often contain genes responsible for many valuable agronomic phenotypes, such as insect resistance and improved nutritional quality (Rubiales et al., 2014; Zhang et al., 2015).

Chinese cabbage is the main leafy vegetable available in North China in the winter due to its high yield and ease of storage. In the current study, we utilized a purple Chinese cabbage germplasm with excess anthocyanin accumulation in its leaves (Sun et al., 2006). In constructing this Chinese cabbage (*Brassica rapa* L. ssp. *pekinensis*) germplasm, the first hybridization was carried out between the projected recipient *B. rapa* (AA genome of the U-triangle, 2*n* = 20) (Nagaharu, 1935) and the anthocyanin candidate donor *B. juncea* (AABB genome of the U-triangle, 4*n* = 36). Previous HPLC analysis showed significantly increased anthocyanin levels in purple cabbage compared to its green *B. rapa* parent. Purple *B. juncea* and purple cabbage both contain the cyanidin-type of anthocyanidin, but with slightly different modification groups (Zhang et al., 2014). Anthocyanins contribute greatly to the quality of horticultural crops by affecting their colors and health-promoting properties. High dietary intake of foods rich in anthocyanins can help reduce cancer risks (Thomasset et al., 2009). Clarifying the mechanisms underlying the formation of purple cabbage progeny will greatly facilitate breeding.

Anthocyanins, the most prominent class of flavonoids, which are widespread in nature, are implicated in protecting vegetative organs from biotic and abiotic stress (Steyn et al., 2002). Structural genes in the anthocyanin biosynthesis pathway were identified and characterized in early studies of *Zea mays, Antirrhinum majus, Petunia hybrid*, and *Arabidopsis thaliana* (Winkel-Shirley, 2001; Koes et al., 2005). Anthocyanin biosynthesis is primarily regulated at the transcriptional level. Studies in *A. thaliana* revealed the presence of the MYB-bHLH-WD40 (M-B-W) ternary transcriptional activation complex (Zhang et al., 2003; Baudry et al., 2004), which has subsequently been identified in a number of species (Feller et al., 2011). The transcription factors R2R3-MYB and bHLH can bind to specific *cis*-elements in promoters of structural genes (Lang et al., 2010). While the levels of MYB and bHLH differed from cell types and fluctuate in changing environments (Albert et al., 2011), the transcription factor WD40 always maintained at the same level (Morita et al., 2006). Some MYB and bHLH proteins are autoregulated (Baudry et al., 2006; Espley et al., 2009), and bHLH can be regulated by MYB (Baudry et al., 2004; Zhu et al., 2015). In seedlings and leaves of *Arabidopsis*, the R2R3-MYB proteins PAP1, PAP2, MYB113, and MYB114, the bHLH proteins TT8, EGL3, and GL3 and the WD40 protein TTG1 are involved in the upregulation of anthocyanin biosynthesis (Gonzalez et al., 2008). When *PAP1* was overexpressed in *Arabidopsis*, the anthocyanin content dramatically increased (Tohge et al., 2005; Rowan et al., 2009), as was the case for its homologs in *Brassica* (Chiu et al., 2010). Additional R2R3-MYB homologs are involved in anthocyanin biosynthesis in *Solanum lycopersicum* (*ANT1*) (Mathews et al., 2003), *Ipomoea batatas* (*IbMYB1*) (Mano et al., 2007), *Malus domestica* (*MdMYB10*) (Espley et al., 2009), and *Citrus sinensis* (*Ruby*) (Butelli et al., 2012).

Regulatory mechanisms for anthocyanin biosynthesis in many species, especially in the model plant *Arabidopsis*, are relatively well-understood, and they should help in clarifying the mechanisms underlying the formation of the anthocyanin overaccumulation phenotype of the parental purple *Brassica juncea*. However, the situation is complicated in the case of purple *B. rapa* obtained from introgressive hybridization. We need to solve not only the problem of finding the candidate genes but also the flow patterns of genetic materials from the donor genome to the recipient. Confirmation of the flowed components will also help in the search for the candidate genes in the progeny. In the current study, we performed RNA-seq of an introgression line to obtain information.

Transcriptome sequencing techniques, such as microarray analysis and RNA-seq, have led to breakthroughs in understanding the genetic mechanisms of metabolism pathways at the transcriptional level. RNA-seq provides more information on both known and unknown transcripts, and it is more suitable for data mining of a known genome with generally unknown exogenous segments containing candidate genes responsible for the interesting traits. However, use of the current pipelines has been restricted to species with (Trapnell et al., 2012) or without (Grabherr et al., 2011) a whole-genome reference sequence. For introgressive hybridization, the use of a pipeline with a reference sequence (with-reference pipeline) often results in the loss of exogenous transcripts, while the use of a pipeline without a reference sequence (without-reference pipeline) often leads to the loss of genome location and junction information. Using a novel bioinformatic analysis strategy that combines with-reference and without-reference pipelines, we aimed to determine the candidate factors responsible for the formation of the purple leaf trait in donor *B. juncea* and hybrid *B. rapa* that flowed between different genomes. During breeding, hybridization processes often occur between a projected crop species and its relatives with interesting traits to genetically improve crop quality. This introgression line transcriptome-based method will be useful for mining candidate genes of interesting traits in different crops and horticultural species.

## Materials and methods

### Plant materials and sample collection

**Figure 1:**
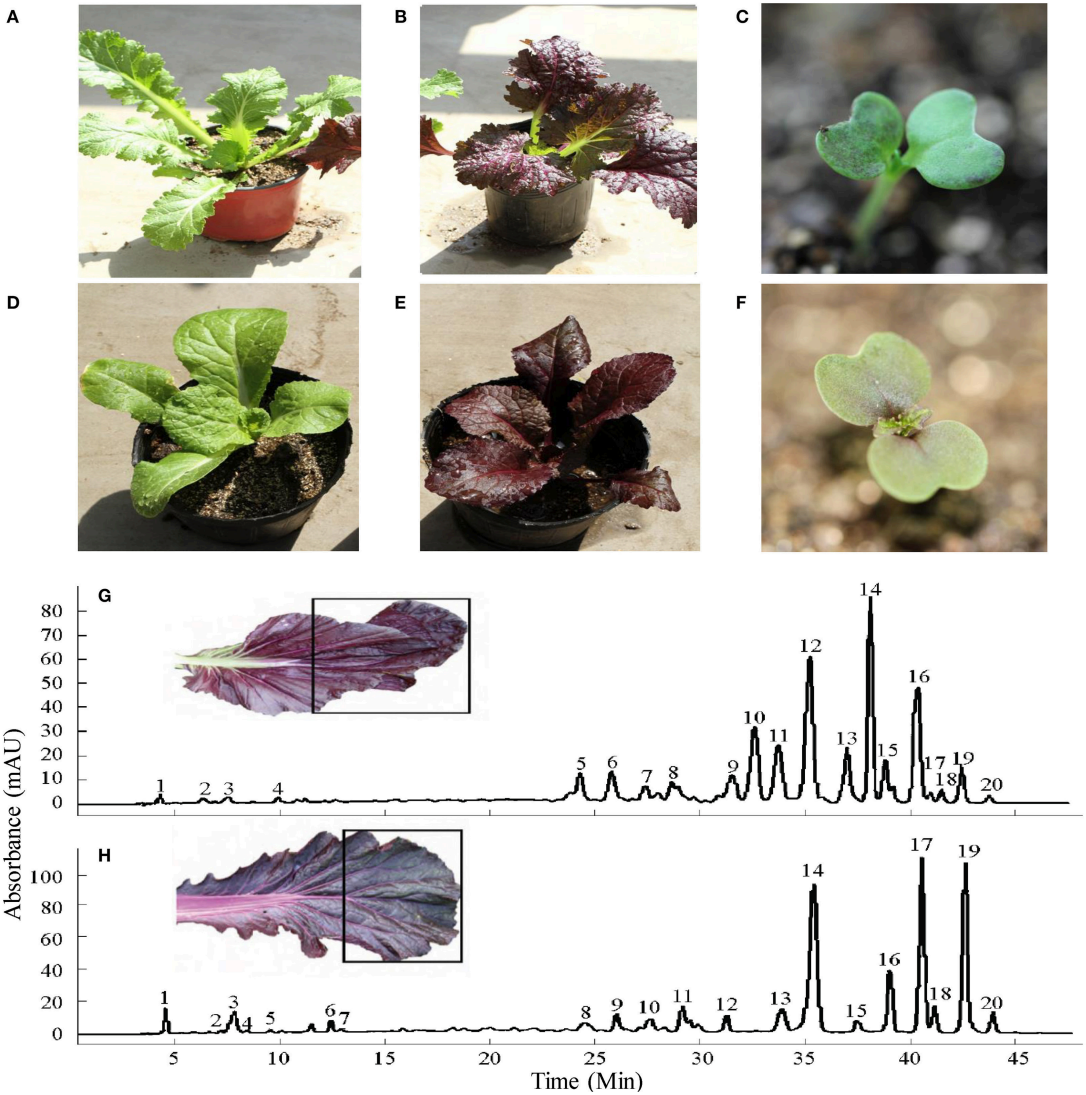
**Young plants of green ***B. juncea*** (A) and donor purple ***B. juncea*** (B) cotyledons of purple ***B. juncea*** (C) young plants of recipient green ***B. rapa*** (D) and introgression line purple ***B. rapa*** (E) cotyledons of purple ***B. rapa*** (F)**. HPLC chromatograms for the anthocyanins of purple *B. juncea* **(G)** and purple *B. rapa* **(H)** extracts recorded at 530 nm (peak numbers refer to the anthocyanins listed in Table 1). The black boxes on leaves show the sampling sections. Modified from Zhang et al. (2014).

The *B. rapa* introgression line (Figure 1E) with dark-purple leaves was derived from a cross between *B. juncea* “Hunan Qianyang” (donor, AABB; 2*n* = 36) and *B. rapa* “Charming Yellow” (recipient, AA; 2*n* = 20) by using the embryo rescue technique (Sharmal et al., 1996). Purple *B. juncea* is a local variety of Hunan Province, China, and it originated by natural mutation. To clarify the inheritance pattern of purple *B. juncea*, we crossed purple *B. juncea* with a green cultivar and grew F_2_ segregation populations (*n* > 200) for 2 years in an open field. The chi-square showed that the F_2_ segregation ratio of purple to green individuals was 3:1. Heterologous hybrids of the donor and recipient with the purple phenotype were selected to backcross with the recipient green *B. rapa* for one generation, followed by self-crossing for three generations, after which the leaf color trait and chromosome number (2*n* = 20) of the offspring became stable (Li and Zhang unpublished data). To collect samples for transcriptome sequencing, purple *B. rapa* introgression line (maternal parent) and green *B. rapa* (paternal parent) individuals were crossed to construct the F_1_ generation and F_2_ segregation population. The plants used for RNA-seq, high-performance liquid chromatography with mass spectrometry (HPLC-MS), and PCR were grown in an open field in Beijing, China, at a temperature of 10 to 15°C.

For RNA-seq, cotyledons of two purple (P1 and P2) and two green (G1 and G2) Chinese cabbages were randomly chosen from the F_2_ population and used to construct an mRNA-seq library with the TruSeq RNA Sample Preparation Kit (Illumina) and insert sizes of 200 ± 25 bp for each sample and was sequenced using the Illumina HiSeq™2000 platform. All RNA-seq reads and *de-novo* assembled transcripts have been submitted to the NCBI SRA and TSA databases (http://www.ncbi.nlm.nih.gov/) under BioProject 312129.

**Table 1:** **Anthocyanin identified in leaves of the purple ***B. juncea*** and introgression line purple ***B. rapa*****.

***B. juncea***					
**Peak No**^a^	**RT**^b^ **(min)**	**Fragments (*****m/z*****)**	**Tentative Anthocyanin**	**Content (**μ**g/g FW**^c^**)**	
				**Purple**	**Green**
1	4.54	303	Delphinidin 3-glucoside	0.34	nd^d^
2	6.57	1137/933/535/287	Cyanidin 3-*p*-coumaroylmalonylsophoroside-5-feruloylglucoside	0.37	nd
3	7.76	535/287	Cyanidin 3-malonylsophoroside-5-glucoside	0.52	nd
4	10.15	1176/993/787/535/287	Cyanidin 3-caffeoylferuloylsophoroside-5-malonylglucoside	0.28	nd
5	24.48	1176/993/787/535/287	Cyanidin 3-caffeoylferuloylsophoroside-5-malonylglucoside	22.07	nd
6	25.98	757/535/287	Cyanidin 3-*p*-coumaroylmalonylglucoside-5-glucoside	25.1	nd
7	27.61	757/577/449/287	Cyanidin 3-*p*-coumaroylsophoroside-5-glucoside	6.54	nd
8	29.12	787/449/287	Cyanidin 3-feruloylsophoroside-5-glucoside	5.26	nd
9	31.71	757,535,287	Cyanidin 3-*p*-coumaroylmalonylglucoside-5-glucoside	26.07	nd
10	32.77	1176/993/787/535/287	Cyanidin 3-caffeoylferuloylsophoroside-5-malonylglucoside	78.87	nd
11	33.89	757/535/287	Cyanidin 3-*p*-coumaroylmalonylglucoside-5-glucoside	68.04	nd
12	35.4	787/535/287	Cyanidin 3-feruloylmalonylsophoroside-5-glucoside	151.89	nd
13	37.16	1167/963/535/287	Cyanidin 3-*p*-coumaroylsinapoylsophoroside-5-malonylglucoside	42.73	nd
14	38.27	1197/993/535/287	Cyanidin 3-sinapoylferuloylsophoroside-5-malonylglucoside	135.15	nd
15	38.99	1167/963/535/287	Cyanidin 3-*p*-coumaroylsinapoylsophoroside-5-malonylglucoside	23.22	nd
16	40.54	1197/993/535/287	Cyanidin 3-sinapoylferuloylsophoroside-5-malonylglucoside	114.47	nd
17	41.13	1137/933/535/287	Cyanidin 3-*p*-coumaroylferuloylsophoroside-5-malonylglucoside	0.7	nd
18	41.65	787/535/449/287	Cyanidin 3-feruloylmalonylsophoroside-5-glucoside	0.91	nd
19	42.62	1167/963/535/287	Cyanidin 3-*p*-coumaroylsinapoylsophoroside-5-malonylglucoside	15.93	nd
20	43.92	1137/993/535/287	Cyanidin 3-*p*-coumaroylferuloylsophoroside-5-malonylglucoside	0.58	nd
***B. rapa***					
**Peak No**.	**RT (min)**	**Fragments (*****m/z*****)**	**Tentative Anthocyanin**	**Content (**μ**g/g FW)**	
				**Purple**	**Green**
1	4.56	303	Delphinidin 3-glucoside	3.33	nd
2	7.18	465/303	Delphinidin 3,5-glucoside	0.24	nd
3	7.81	535/287	Cyanidin 3-malonylsophoroside-5-glucoside	16.46	nd
4	8.42	449/287	Cyanidin 3,5-diglucoside	0.34	nd
5	9.53	479/317	Petundin 3,5-diglucoside	0.36	nd
6	12.41	787/535/287	Cyanidin 3-feruloylmalonylsophoroside-5-glucoside	0.97	nd
7	12.89	757/535/287	Cyanidin 3-*p*-coumaroylmalonylsophoroside-5-glucoside	0.41	nd
8	24.51	773/535/287	Cyanidin 3-caffeoylmalonylsophoroside-5-glucoside	5.24	nd
9	26.02	787/535/287	Cyanidin 3-feruloylmalonylsophoroside-5-glucoside	9.54	nd
10	27.62	1167/963/535/287	Cyanidin 3-feruloylmalonylsophoroside-5-feruloylglucoside	8.04	nd
11	29.13	787/449/287	Cyanidin 3-feruloylsophoroside-5-glucoside	15.1	nd
12	31.24	1176/993/787/535/287	Cyanidin 3-caffeoylferuloylsophoroside-5-malonylglucoside	7.46	nd
13	33.87	757/535/287	Cyanidin 3-*p*-coumaroylmalonylglucoside-5-glucoside	23.6	nd
14	35.39	787/535/287	Cyanidin 3-feruloylmalonylsophoroside-5-glucoside	180.17	nd
15	37.44	979/449/287	Cyanidin 3-sinapoyl-*p*-coumaroplsophoroside-5-glucoside	5.91	nd
16	39.02	1167/963/535/287	Cyanidin 3-diferuloylsophoroside-5-malonylglucoside	42.38	nd
17	40.55	1197/993/535/287	Cyanidin 3-sinapoylferuloylsophoroside-5-malonylglucoside	134.71	nd
18	41.13	1137/933/535/287	Cyanidin 3-*p*-coumaroylferuloylsophoroside-5-malonylglucoside	13.61	nd
19	42.61	1167/963/535/287	Cyanidin 3-diferuloylsophoroside-5-malonylglucoside	126.89	nd
20	43.92	1137/933/535/287	Cyanidin 3-*p*-coumaroylferuloylsophoroside-5-malonylglucoside	9.27	nd

a Peak number corresponds to elution order by HPLC analysis in Figure 1.

b Retention time.

c Fresh weight.

d Not detected.

Modified from Zhang et al. (2014).

For HPLC-MS analysis, the tip of the blades were cut from young leaves of purple *B. juncea* and purple *B. rapa*. For PCR amplification of DNA sequences of predicted transcripts, young leaves of *B. rapa* (green, recipient), *Brassica nigra* (green), *B. juncea* (purple, donor), *B. rapa* (purple, introgression line), *B. rapa* (green), and purple and green individuals from F_2_ segregation of the *B. rapa* introgression line and green *B. rapa* were collected.

For cold and high light treatments, all the purple and green *B. juncea* and purple *B. rapa* individuals were grown till 1 month of age in a greenhouse and then transferred to an artificial climate incubator (MGC-250P; Shanghai Yiheng Technical Co., Ltd). The plants were initially grown under 16/8-h light/dark cycles for 5 days, with a light intensity of 4000 lx and a temperature of 25°C. At the end of this period, young leaves of the green and purple *B. juncea* lines (7 cm in length) were collected. Green *B. juncea* samples were used as the control for purple *B. juncea*, and the two purple lines were used as the control for the stress environmental treatments. Then, light intensity and temperature parameters were changed to 12,000 lx and 10°C, respectively, for the same period of 5 days, and leaves of the same size were selected for the cold and high light treatments. Three biological replicates were collected at each sampling time.

### HPLC-MS analysis

The leaf samples were extracted in 60% ethanol aqueous solution (pH value adjusted to 3; solid:liquid ratio of 1:20) at 50°C for 2 h and then filtered through a membrane filter (0.22 μm). The extract was injected into a C_18_ column (4.6 × 150 mm, 5 μm; Waters XBridge) mounted on an analytical HPLC-MS system (Agilent 1200 series, Ion Trap 6310). Elution was performed using mobile phase A (5% formic acid aqueous solution) and mobile phase B (acetonitrile). Detection was performed at 530 nm, and the column oven temperature was set at 25°C. The flow rate was 0.8 mL/min. The gradient program was as follows: 0–15 min, 10–13% B; 15–45 min, 13–20% B; 45–50 min, 20–23% B; and 50–55 min, 23–100% B. Quantification of the different anthocyanins was based on peak areas and calculated as equivalents of the external standards; cyanidin 3,5-diglucoside was used as the reference standard (Extrasynthese). The mass spectrometer conditions were as follows: ESI interface; nebulizer, 50 psi; dry temperature, 350°C; scan range, 100–1500 m/z; and nitrogen flow rate, 12 L/min.

### Transcriptome analysis

**Figure 2:**
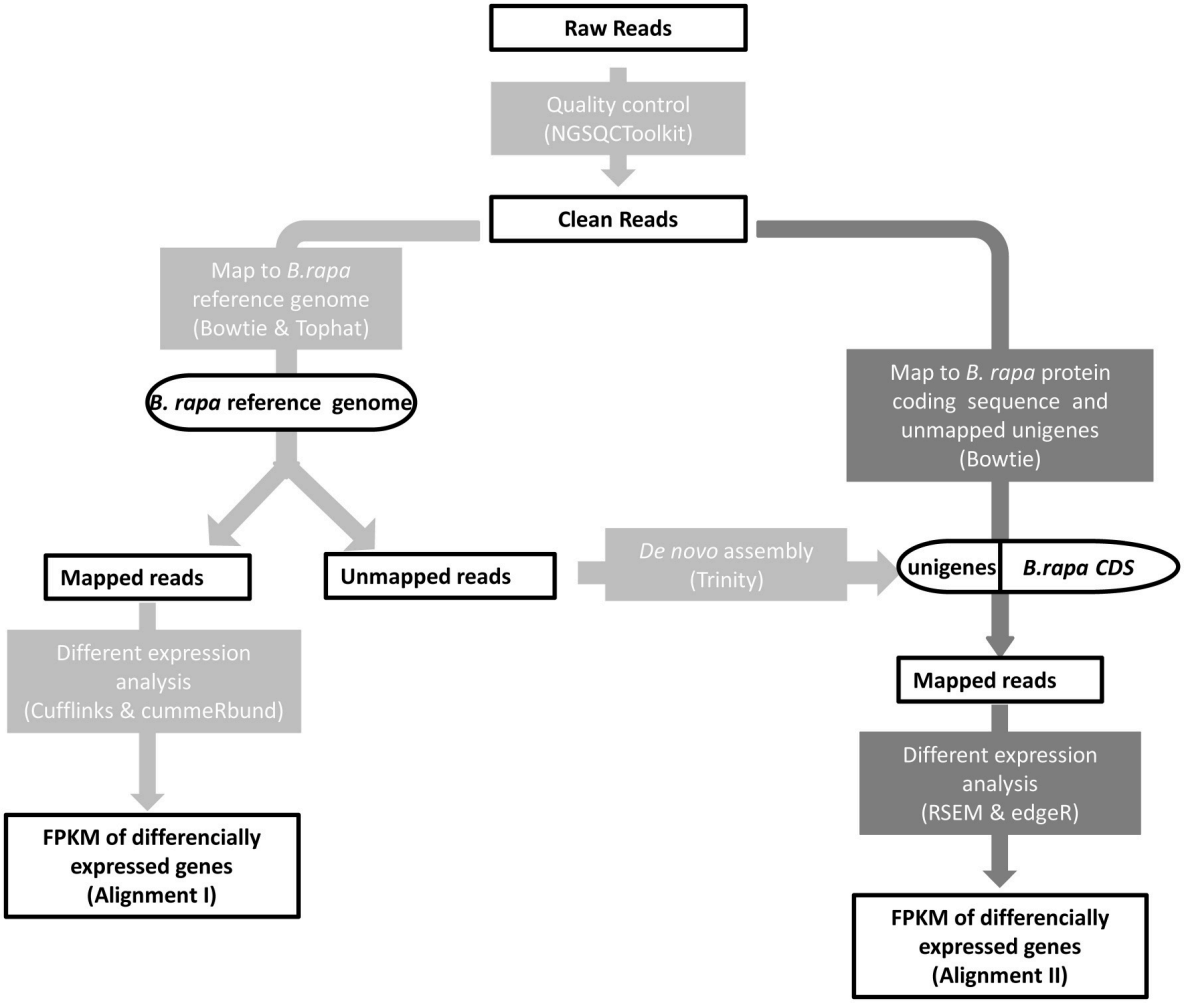
**Mapping strategy**. Alignment I (light gray): reads mapping to the *B. rapa* reference genome; Alignment II (dark gray): reads stem from the unmapped reads of Alignment I mapping to the *B. rapa* transcript sequences and *de novo* assembled unigenes.

The *B. rapa* reference genome v1.5 sequence and the 41,019 reference transcripts were downloaded from the BRAD database (http://brassicadb.org/) (Cheng et al., 2011). As shown in Figure 2, two successive alignments of reads were performed for DEG analysis. The first step was alignment of the reads to the *B. rapa* reference genome. To process the raw reads, NGS QC Toolkit v2.3.3 (Patel and Jain, 2012) was used to discard the pair-end reads containing ambiguous Ns or low quality bases (PHRED-like score <20) exceeding 20%. The first unstable 10 bp of filtered reads were trimmed. Clean reads of four samples were mapped to the *B. rapa* reference genome sequence using TopHat v2.0.9 (Trapnell et al., 2009) with default settings. After duplicates were removed using SAMtools v0.1.19 (Li et al., 2009), the accepted reads were assembled according to the *B. rapa* reference general feature format files, and the abundance of each sample was estimated using Cufflinks (v2.2.1) (Trapnell et al., 2010). DEGs were extracted using cummeRbund v2.12.0 (Goff et al., 2013) with a false discovery rate (FDR ≤ 0.001) and |log2FC| ≥ 1 and were displayed using Circos v0.66 (Krzywinski et al., 2009). The unmapped reads were transformed back to fastq format with bam2fastq v1.1.0 (http://www.hudsonalpha.org/gsl/information/software/bam2fastq) for subsequent *de novo* assembly using Trinity r20140717 (Grabherr et al., 2011). The ORFs were predicted and redundancy was removed using EMBOSS v6.6.0 (Rice et al., 2000) and Cd-hit v4.6.1 (Li and Godzik, 2006), respectively.

Second, clean reads were aligned to the merged transcript sequences of 41,019 reference *B. rapa* genes and 27,286 newly obtained unigenes from the *de novo* assembly of unmapped reads. The mapping statistics of the two alignments, Alignment I and Alignment II, are shown in Supplemental Table 4. Only uniquely mapped reads were reserved. RSEM v1.2.8 (Li and Dewey, 2011) and edgeR v3.12.0 (Robinson et al., 2010) were used to distinguish the DEGs. The same thresholds of FDR ≤ 0.001 and |log2FC| ≥ 1 or more were used to judge the significance of differences and to identify DEGs. Annotation of these transcripts was performed with MapMan (Thimm et al., 2004) using homologous *A. thaliana* genes in TAIR10 (Lamesch et al., 2012). The conserved domain annotation was performed using CCD (Marchler-Bauer et al., 2015) in NCBI.

### Multiple sequence alignment and phylogenetic analysis

*R2R3-MYB* gene families were obtained from the *B. rapa* genome using the BLAST algorithm v2.2.28+ (Altschul et al., 1990) and HMMer v3.0 (Eddy, 2009) and were visually verified. Anthocyanin-related *R2R3-MYB* homologous protein sequences were obtained from GenBank. A multiple sequence alignment was assembled using MAFFT v7.266 (Katoh et al., 2002) and manually adjusted. The conserved domain (nucleotide-binding site) sequences of the R2R3-MYB proteins were extracted to construct a neighbor-joining tree in MEGA5 (Tamura et al., 2011) with 1000 bootstraps.

### Sequence amplification and real-time quantitative PCR

DNA was isolated from leaves by the CTAB method and diluted to ~500 ng/μl. Full-length gene sequences or fragments were amplified using Ex Taq® (Takara) DNA polymerase. Total RNA was isolated from young leaves using a Trizol® Reagent Kit (Invitrogen) according to the manufacturer's instructions. Subsequently, the RNA was reverse transcribed using a TransScript® One-Step gDNA Removal and cDNA Synthesis SuperMix Kit (TransGen Biotech Co., Ltd.). Real-time quantitative PCR (qPCR) was performed in a MX3000P qPCR system (Agilent). The PCR mixture (final volume of 20 μL) contained 10 μL SYBR® Premix Ex Taq™ (Takara), 0.4 μL ROX Reference Dye (Takara), 0.2 μM of each gene-specific forward and reverse primer and ~50 ng of cDNA. The following thermal profile was used: 45 cycles of 95°C for 5 s, 56°C for 10 s and 72°C for 10 s. The results were analyzed using the 2^−ΔΔ*C*T^ method (Livak and Schmittgen, 2001), with the stable housekeeping gene *eEF1B*α*2* (*Bra002241*) as the internal control (Tong et al., 2013). Primers used in the above experiments are listed in Supplemental Table 5.

## Results

### Alignment of reads to the *B. rapa* reference genome and differentially expressed gene (DEG) analysis reveal up-regulated genes in the anthocyanin pathway

The *B. rapa* introgression line (Figure 1E) used in this study, with dark-purple leaves, was derived from a cross between purple *B. juncea* and green *B. rapa* by using the embryo rescue technique (see Materials and Methods). The purple phenotype of the donor plant *B. juncea* was controlled by a single gene with dominant inheritance, as determined by the hybridization and F_2_ segregation ratio (purple:green = 3:1) between purple *B. juncea* (Figure 1B) and green *B. juncea* (Figure 1A). The leaf blades of the purple *B. rapa* were darker than that of the donor purple plant; the leaf veins of *B. rapa* were purple, while the leaf veins of *B. juncea* were green (Figures 1B,E). Pigments were differentially distributed in the cotyledons: Dark purple color was scattered in *B. juncea* and gathered near the veins in *B. rapa*. (Figures 1C,F). We used the purple *B. rapa* and green *B. rapa* (Figure 1D) as the parents to obtain an F_2_ segregation population. Purple and green individuals in the F_2_ population were selected to perform chemical component and transcriptome analyses. Anthocyanin contents and components of the donor purple *B. juncea* and introgression line purple *B. rapa* were extracted for the HPLC-MS analysis (Figures 1G,H; Table 1), as described in our previous study (Zhang et al., 2014). The analysis revealed that the purple individuals of *B. juncea* and *B. rapa* all accumulated higher contents of anthocyanins than the green ones; both had cyanidin-type anthocyanins as the main components, but differences existed in the modification of the acyl groups (Table 1).

Cotyledons of two purple and two green Chinese cabbage from the F_2_ segregation population were collected for RNA sequencing. Because the main genetic background of this Chinese cabbage introgression line is *B. rapa*, we first mapped the clean reads to the genome sequence of *B. rapa* cultivar “Chiifu” and identified DEGs, as shown in the light-gray pipeline on the left side of Figure 2. Under a threshold of FDR ≤ 0.001, |Log2FC| ≥ 1 and fragments per kilobase of transcript per million (FPKM) (Mortazavi et al., 2008) mapped reads values > 10 in both sample sets, we determined 930 significant DEGs (Supplemental Table 1), including 389 up-regulated and 541 down-regulated.

**Figure 3:**
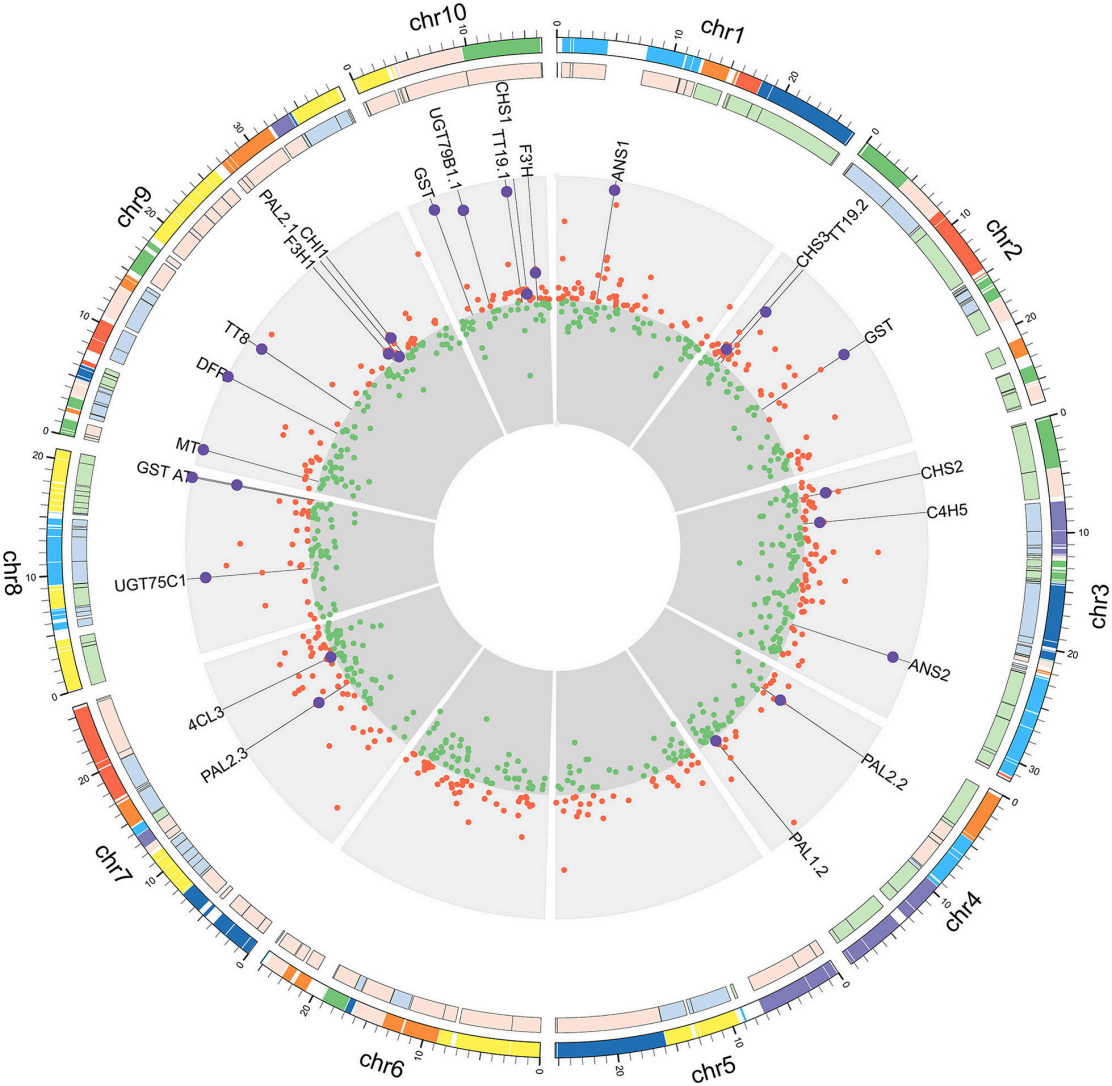
**DEGs from reads mapped to the ***B. rapa*** genome**. From the outer ring to the center: genome blocks designated by different colors (Cheng et al., 2013); degree of gene density designated by different colors (Cheng et al., 2014); Log2FC values of up-regulated genes (red circle, outward orientation); Log2FC values of down-regulated genes (green circle, inward orientation). The values in both up- and down-regulated regions were set to the highest |log2FC| value. Larger purple circles represent anthocyanin-related genes.

Among the up-regulated genes, almost all of the structural anthocyanin pathway genes (Guo et al., 2014) were found, as they had significantly higher fold-changes than average (Figure 3). These anthocyanin pathway genes were scattered among the chromosomes, and their expression levels did not show any correlation with genome blocks or degrees of gene density (Cheng et al., 2014). This result indicates that regulatory genes or more upstream mechanisms play a major role in coordinately regulating the entire pathway.

The formation and amplification of the ternary transcriptional activation complex are prerequisites for the transcription of structural genes in the anthocyanin pathway (Zhu et al., 2015). Thus, except for *WD40*, which is constitutively expressed, at least one *bHLH* and one *R2R3-MYB* should be up-regulated in the purple line. However, only one regulatory gene, *Bra037887* (*bHLH*), was significantly up-regulated in this line, and we did not find any up-regulated *R2R3-MYB* genes among the DEGs in the *B. rapa* genome, suggesting that some information was missing. Therefore, to avoid missing any information, we focused on unmapped reads.

### The *de novo* assembly of unmapped reads and mining of a new set of candidate genes

**Figure 4:**
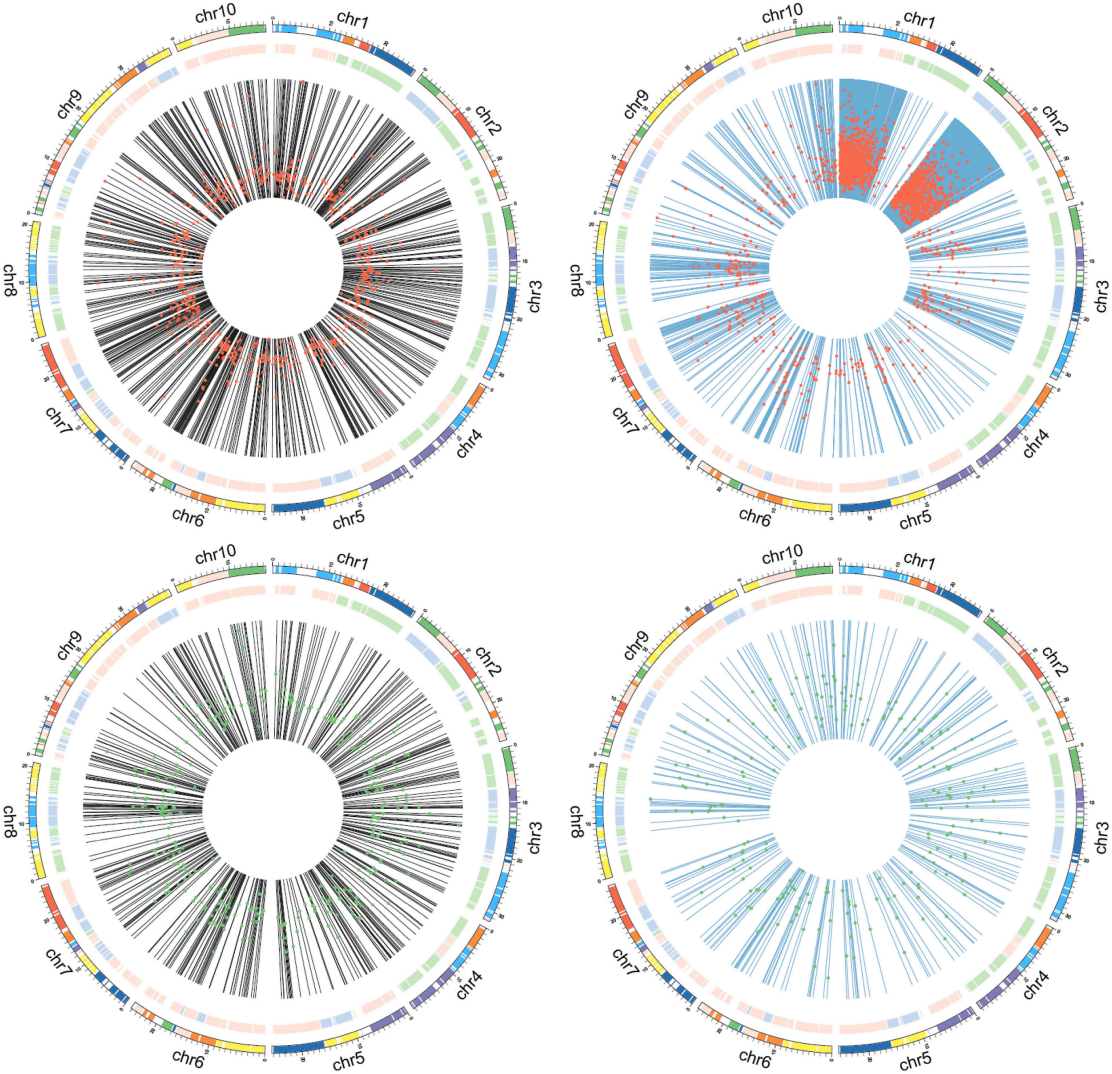
**Chromosomal locations of differentially expressed transcripts**. Black lines show the chromosomal locations of *B. rapa* genes, blue lines show the chromosomal locations of *B. rapa* genes most similar to unigenes; red dots represent upregulation, with Log2FC values increasing from the inner to outer regions; green dots represent downregulation, with Log2FC values increasing from the inner to outer region.

To obtain additional information, we mined unmapped reads. After *de novo* assembly and identification of unique ORFs, 27,286 unigenes were discovered. We merged these unigenes with 41,019 *B. rapa* gene-coding sequences and aligned the clean reads from four samples to this new chimeric database (Figure 2, dark gray pipeline). Under the threshold of FDR ≤ 0.001 and |Log2FC| ≥ 1, a total of 2996 differentially expressed transcripts (Supplemental Table 2) were identified, including 2031 *de novo* assembled unigenes. By comparing these unigenes with their *B. rapa* homologs, were determined that their similarity values mainly ranged from 90 to 95%. Considering the homologous recombination events during hybridization, and the source of those *de novo* assembled transcripts, we thought that chromosomal locations of transcript-coding genes might reflect some specific patterns. So we divided the 2996 transcripts into four groups, including 580 up-regulated *B. rapa* genes, 385 down-regulated *B. rapa* genes, 1814 up-regulated unigenes and 217 down-regulated unigenes, and we displayed the genome locations of *B. rapa* genes or the best *B. rapa* genes hits of unigenes. An interesting pattern emerged, that is, the 1814 up-regulated unigenes were clustered in two segments on chromosome 1 and 2, while the three other groups were not (Figure 4). These results suggest that a large segment may have been imported from the exogenous genome to the *B. rapa* AA genome.

**Figure 5:**
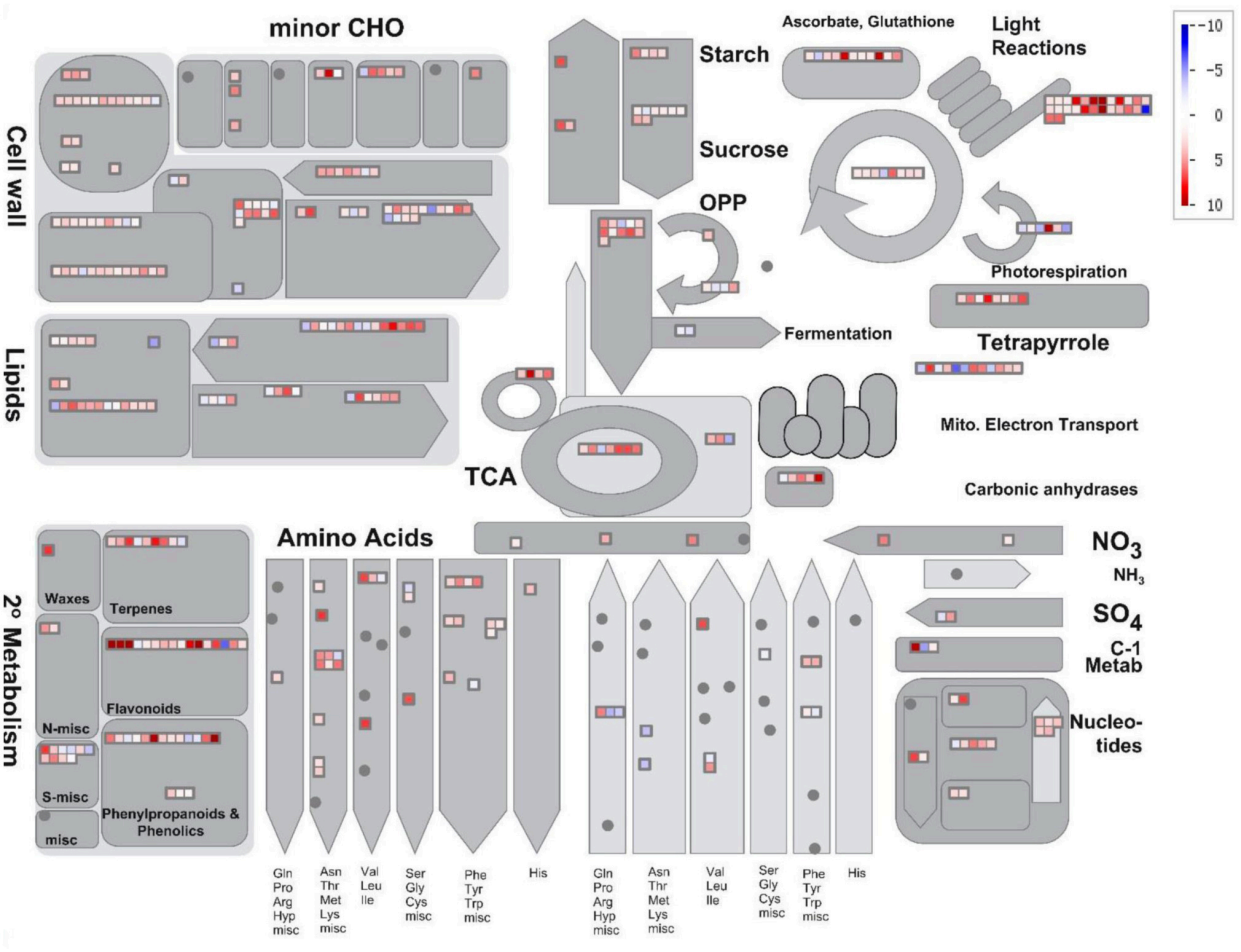
**Overview of changes in metabolic-related gene expression in anthocyanin-overaccumulating leaves**. DEGs were binned to MapMan functional categories. Log2FC values of up- and down-regulated transcripts are shown in red and blue, respectively.

**Figure 6:**
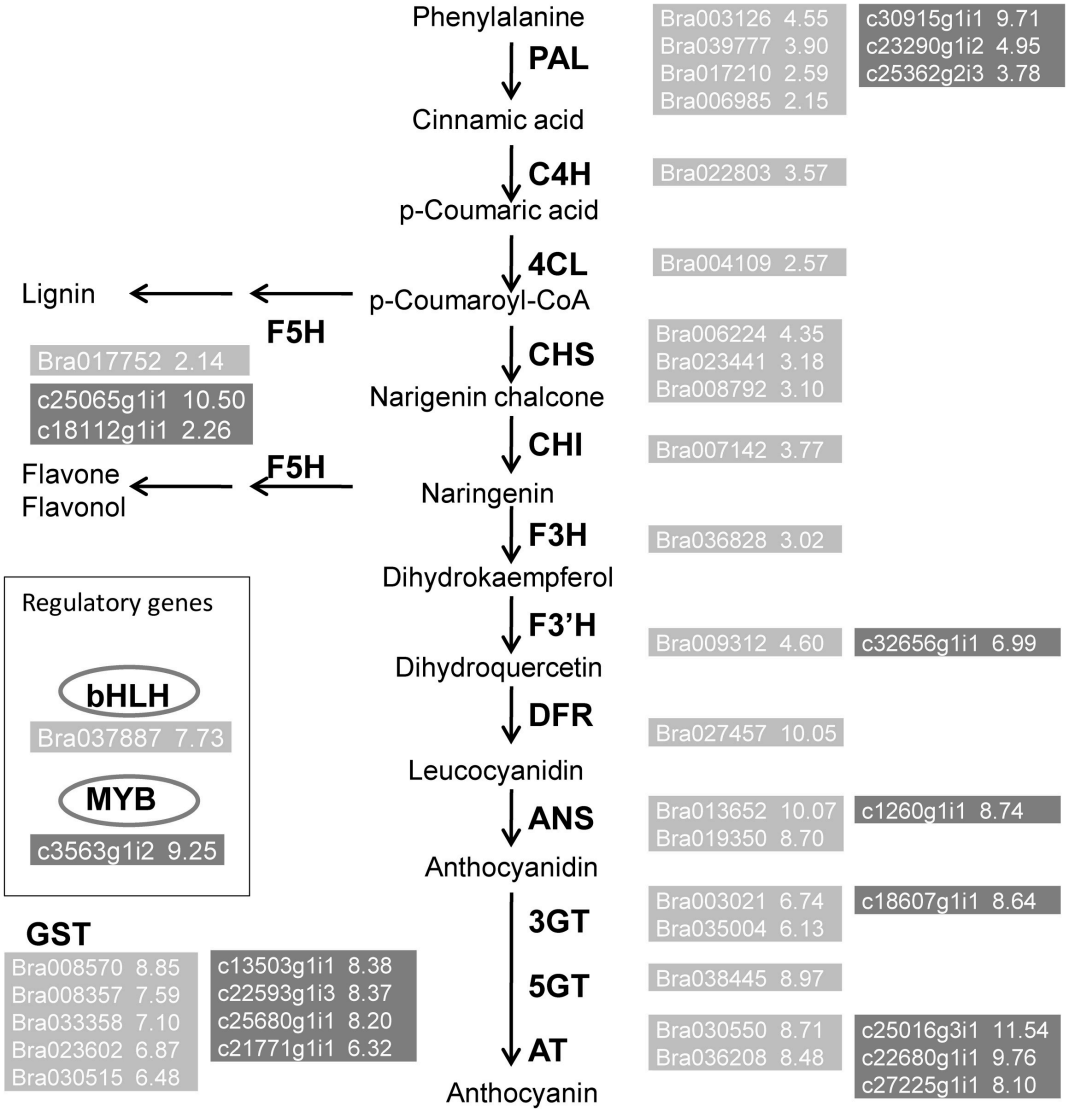
**Up regulated genes belong to the anthocyanin biosynthesis pathway**. Transcripts obtained from Alignment I (light gray) and Alignment II (dark gray). Numbers beside gene names represent up-regulated Log2FC values.

Figure 5 shows a depiction of the up- and downregulation of the transcripts constructed using MapMan software. Several significantly differentially expressed pathways were distinguished by the Wilcoxon Rank Sum test (probability < 0.05), including hormone metabolism, protein, tricarboxylic acid cycle, minor carbohydrate metabolism, signaling and secondary metabolism. When the DEGs were sorted by the Log2FC values of transcripts, anthocyanin pathway genes, from *phenylalanine ammonia lyase* to *glutathione-S-transferase*, were clearly up-regulated synergistically. Supplemental Table 3 lists the Log2FC values of anthocyanin pathway genes originating from the two alignments. The Log2FC values between these alignments had a Spearman correlation coefficient of 0.99, indicating the consistency of the results achieved using the two different methods. However, there were additional homologous transcripts in the unmapped reads (Figure 6, dark gray background). These anthocyanin structural genes with a non-AA genome origin may contribute to the dosage effect responsible for the darker purple appearance of introgression line *B. rapa* compared to their *B. juncea* donor (Figure 1). And the existence of different glycosyltranferases and acyltransferases may cause slight difference of anthocyanin types between purple *B. juncea* and purple Chinese cabbage.

**Figure 7:**
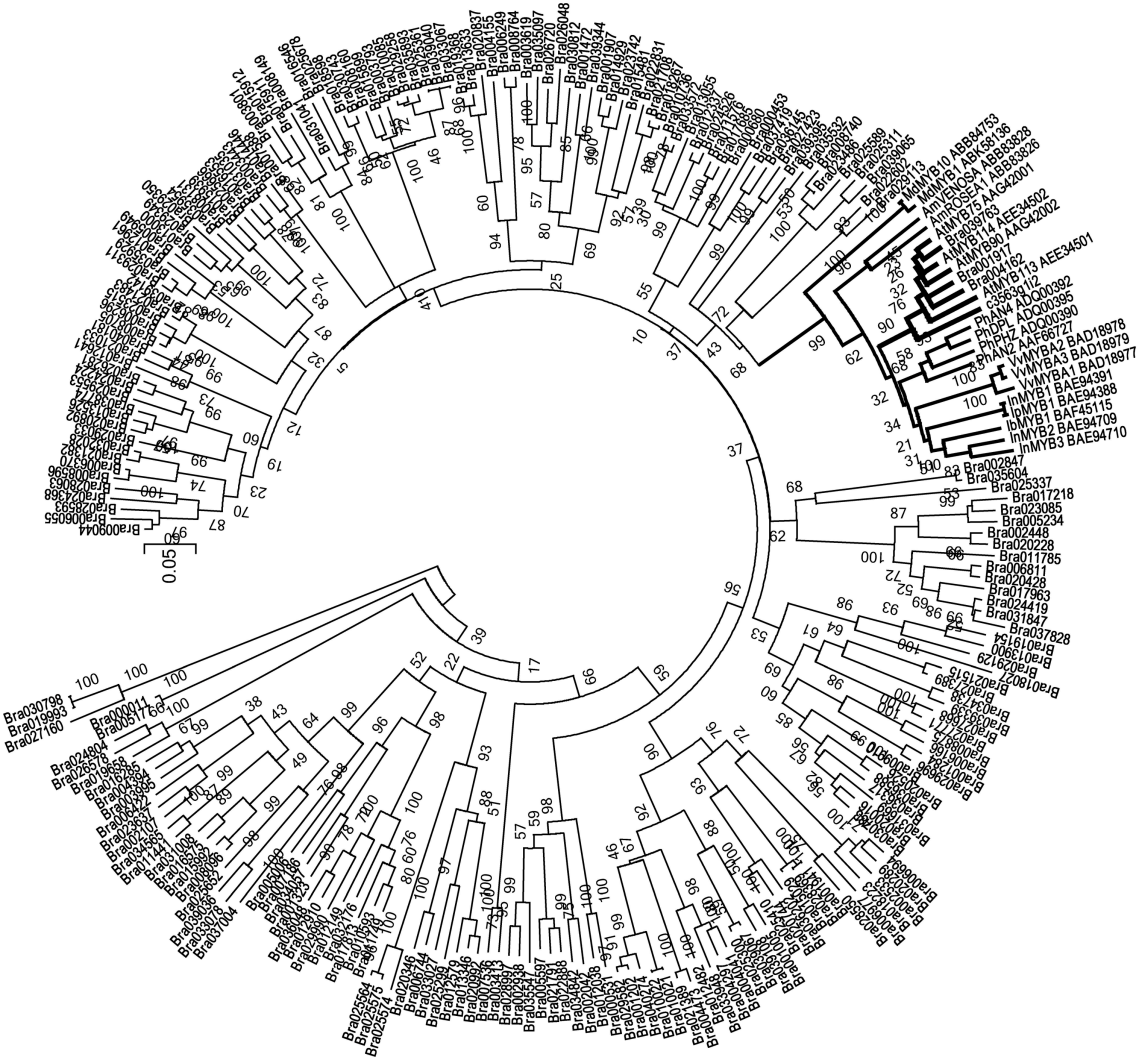
**Neighbor-Joining tree of R2R3-MYB sequences containing c3563g1i2, anthocyanin-related R2R3-MYB (indicated as names in reference, and accession numbers in GenBank) and all of the R2R3-MYB superfamily members of ***B. rapa*****.

More importantly, in the unmapped parts of the reads, we identified an *R2R3-MYB* transcript, *c3563g1i2*. According to the annotation, *c3563g1i2* is the homolog of Arabidopsis *MYB90*, also known as *production of anthocyanin pigment 2* (*PAP2*), implying that its function is related to anthocyanin biosynthesis. We used the protein sequence of c3563g1i2, along with the protein sequences of all R2R3-MYBs in the *B. rapa* genome and anthocyanin-specific R2R3-MYBs in the literature, to construct a neighbor-joining tree (Figure 7). In this phylogenetic tree, c3563g1i2 and its Brassicaceae homologs are clustered together, embedded in a clade consisting of anthocyanin-related R2R3-MYBs of various species and cell types. Thus, c3563g1i2 may represent the R2R3-MYB that participates in the ternary transcriptional activation complex responsible for the anthocyanin overaccumulation phenotype of the *B. rapa* introgression line.

In addition to structural and regulatory genes involved in anthocyanin biosynthesis, genes involved in cold and light reaction pathways, such as homologs of *cold-related 6.6* (*c23142g1i5*) and *photosystem II subunits R* (*c20348g1i2*), were expressed at extremely high levels in purple individuals compared to green individuals in the F_2_ population, as shown in Supplemental Table 2. As it is known that anthocyanin biosynthesis often response to environmental changes, these transcripts may need to be included as candidates, validating their genome sequences at the DNA level and transcriptional expression at the RNA level. Accordingly, the *B. rapa* gene *Bra037887* and the unigenes *c3563g1i2, c23142g1i5*, and *c20348g1i2* were selected for further analysis.

### Experimental verification of candidate genes responsible for the anthocyanin overaccumulation phenotype

**Figure 8:**
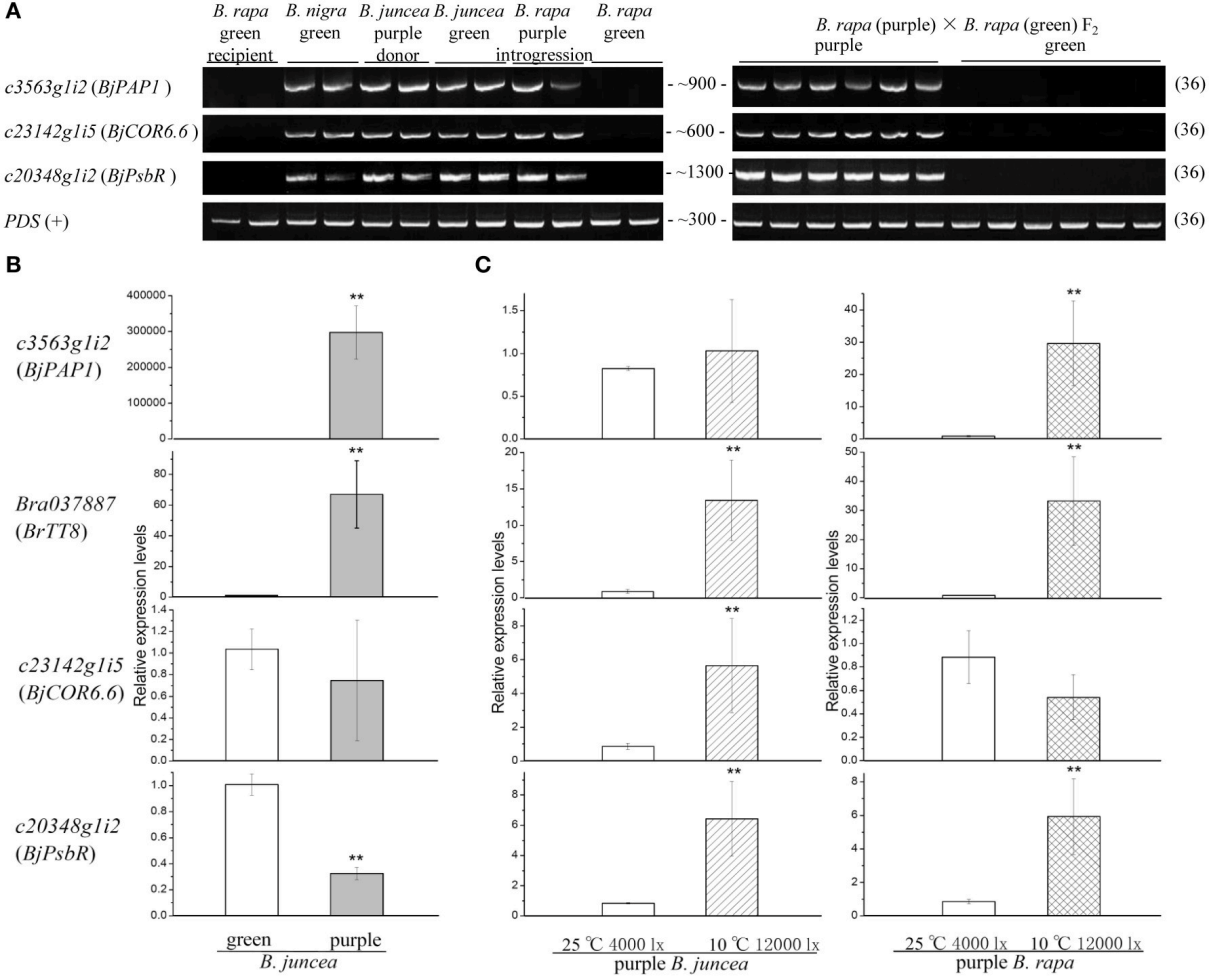
**PCR and qPCR validation of candidate transcripts. (A)** PCR amplification (all 36 cycles) of candidate genes from different lines. *PDS* was used as a positive control. **(B)** Relative expression levels of green and purple *B. juncea* (*eEF1B*α*2* was used as an internal control). **(C)** Relative gene expression levels under cold and high light treatment (*eEF1B*α*2* was used as an internal control). ^**^Means statistically significant.

Bioinformatics analysis of transcripts derived from unmapped reads suggested that B genome components flowed to purple Chinese cabbage. To help confirm this hypothesis, we designed primers for specific sites in unigenes *c3563g1i2, c23142g1i5*, and *c20348g1i2* that can distinguish these genes from their AA genome homologs and performed PCR using DNA samples extracted from the donor plant purple *B. juncea*, recipient green *B. rapa, Brassica* BB genome plant *Brassica nigra* and F_2_ individuals with purple or green leaves. As shown in Figure 8A, bands of *c3563g1i2* (*BjPAP1*), *c23142g1i5* (*BjCOR6.6*), *c20348g1i2* (*BjPsbR*) of the expected sizes could be amplified from purple *B. rapa* and all materials with a *Brassica* B genome, but not from the green *B. rapa* parent or green F_2_ offspring. We confirmed the PCR fragments by Sanger sequencing and found that all had the same sequence as transcripts assembled from RNA-seq reads. These results help confirm the notion that the B genome components flowed to the purple Chinese cabbage genome.

Since, the purple vs. green trait in the *B. rapa* introgression line is not due to the presence or absence of a gene at the DNA level, we did not perform qPCR to compare the transcript abundance in *B. rapa*; instead, we performed qPCR to compare *B. juncea* with purple vs. green leaves. We selected green *B. juncea* varieties grown in the same environment as controls to obtain relative gene expression levels. The anthocyanin pathway regulatory genes *c3563g1i2* and *Bra037887* were significantly up-regulated in purple individual (Figure 8B), especially the *B. juncea PAP1* homolog *c3563g1i2*. This result is consistent with the finding from many previous studies that overexpressing anthocyanin-related *R2R3-MYB* always leads to anthocyanin overaccumulation (Borevitz et al., 2000; Chiu et al., 2010).

During planting, we observed that the color of purple Chinese cabbage leaves often changed to a small extent when the climate changed. To analyze the influence of the environment on gene expression in purple Chinese cabbage, we used temperature and light treatments to observe the behaviors of the selected transcripts (Figure 8C). In previous studies on wild type *Arabidopsis*, low temperature often induced the transcription of anthocyanin regulation genes in a light-dependent manner (Leyva et al., 1995; Soitamo et al., 2008). The light and temperature conditions were set to about 7100 lx (100 μmol·m^−2^·s^−1^) and 4°C, respectively. In a *PAP1* overexpression line, the leaves were dark purple at 22°C (440 μmol·m^−2^·s^−1^) and still purple at 30°C (150 μmol·m^−2^·s^−1^) (Rowan et al., 2009). On the basis of both the reported range and growth circumstances of *Brassica* plants in greenhouses during late autumn (10–25°C and 4000–12000 lx), the treatment parameters in this study were set to a moderate combination of 10°C and 12000 lx, with 25°C and 4000 lx as the controls. In the donor purple *B. juncea*, cold and high light treatments significantly influenced the expression of *Bra037887, c23142g1i5*, and *c20348g1i2*, but not *c3563g1i2*. In purple *B. rapa* individuals of the introgression line, *c3563g1i2, Bra037887*, and *c20348g1i2* were upregulated, but *c23142g1i5* was not. The responses of *c23142g1i5* to external conditions were significant in the original genome, but altered in the new genome. As for *c3563g1i2*, when transferred to another genome, the environmental effect was more apparent. These results reveal the environmental influence on candidate genes and imply that some transcripts exhibit different behaviors during hybridization and gene flow.

According to fold changes, the varying expression of *c3563g1i2* caused by genetic divergence provides the main effect underlying the purple leaf trait. In addition, *c23142g1i5* and *c20348g1i2* are slightly upregulated by the cold and high light in *B. juncea*, suggesting that environmental factors also contribute to the phenotypic presentation. Changes in both DNA and mRNA levels of the candidate genes originating from the donor plants are attributable to the ultimate phenotype of the purple Chinese cabbage.

## Discussion

### Candidate genes flow from the donor's genome to the recipient's genome to influence the hybrid phenotypes in a dosage-dependent manner

By analyzing transcriptomes of Chinese cabbage leaves with purple and green color, we gradually mined candidate genes responsible for anthocyanin overaccumulation trait. Of the mapped *B. rapa* AA genome transcripts examined, we found only the *bHLH* regulatory genes and a greater proportion of structural genes. From the set of unmapped reads, we identified an exogenous *R2R3-MYB* gene and another group of structural genes. PCR and qPCR validation indicated that *c3563g1i2* from the green *B. juncea* and purple *B. juncea* lines did not differ in terms of their protein-coding nucleotide sequences, whereas they exhibited highly different expression levels. As no other *R2R3-MYB* transcript was up-regulated in the purple- vs. green-leaf lines, have the *c3563g1i2* genes or not is the key reason for the purple phenotype in both *B. juncea* and *B. rapa*. The extremely close relationship among species within *Brassica* likely provides compatibility between the M-B-W regulatory machinery and their binding sites in promoters of structural genes originating from the AA or BB genome. Hence, the integrity of the M-B-W regulatory complex and the up-regulation of its members' transcripts are necessary for the formation of the purple trait. Reasons that caused the overexpression of *c3563g1i2* has not been analyzed. Potential differences may exist in *cis* regulatory regions or upstream *trans* factors, or even epigenetic marks (Wang et al., 2013). Moreover, the flowed structural genes may contribute to the content and modification of anthocyanins, which may explain why the purple leaves/cotyledons of the *B. rapa* introgression line are darker than those of the donor plant *B. juncea*, and slight modification difference between them.

In addition, based on RNA-seq sample sets related to anthocyanin phenotypes, analysis of the DEGs revealed some putative factors acting upstream of the M-B-W ternary transcriptional activation complex. Although anthocyanin accumulation is significantly induced after high light and cold treatment, all of the factors in the cascade have not yet been identified. In this study, we first predicted many highly up-regulated transcripts in purple individuals than in green under the same environmental conditions, including factors responding to cold and light, along with hormone and signaling pathways. By PCR and Sanger sequencing, the selected two transcripts *c23142g1i5* and *c20348g1i2* were comfirmed as B genome components. As we investigated a line produced by introgressive hybridization, the large amounts of transcripts from genes encoding cold response and photosystem proteins identified in this study may have been due to the presence of chromosome segments transferred among different genomes on the DNA level. If genes are constitutively and highly expressed in the original genome, they would likely still contribute high levels of transcripts to the new recipient genome, explaining the significant DEGs identified by bioinformatics methods.

We performed qPCR to validate the expression levels of the selected transcripts in the purple *B. juncea* line and purple *B. rapa* introgression line. *B. juncea* homologs of COR6.6 and PsbR were significantly higher under cold and high light than under control conditions. This suggests that these transcripts respond to environmental changes, which might partly explain the small color changes in different climates. However, the presented fold changes were <10. The temperature (10°C) and light (12000 lx) treatments might not reach the strongest stress extent. As for *c3563g1i2*, the treatments did not cause any significant changes. We think this is because the transcriptional expression of *c3563g1i2* in purple *B. juncea* was completely triggered by the genetic effect. Furthermore, we found that some transcripts exhibited divergent behaviors in the original and new recipient genome, for example, *c3563g1i2* and *c23142g1i5*; this suggests a more complicated situation in the background genome. Thus, further analyses of the upstream mechanisms underlying the over-expression pattern of *c3563g1i2* in the purple leaves of *Brassica* and expression behavior changes during transfer between genomes are required.

The identification of sequences with particular differential transcription patterns lays the foundation for further studies, especially in the vegetative tissues of horticultural *Brassica* crops.

### RNA-seq analysis combining the with- and without-reference pipelines will assist in candidate gene mining in introgression lines

Resistance or quality traits of related species provide abundant resources for varietal breeding. During the process of hybridization, the genes responsible for candidate traits are transferred between species. Determining the critical factors and mechanisms underlying this process would provide guidance for future breeding programs. Normally, gene flow caused by recombination or chromosome number variations can be detected using AFLP or SSR markers (Schlötterer, 2004), bulk segregant analysis (Quarrie et al., 1999), or whole-genome resequencing (Bentley, 2006). These methods are used to detect pivotal single nucleotide polymorphisms or insertion-deletions. However, these techniques only offer information at the DNA level, and much more complicated changes occur at the transcriptional or post-transcriptional levels. Therefore, identifying the transcriptomes of tissues with interesting traits using RNA-seq technology represents a more rapid, important supplemental method for elucidating these changes.

RNA-seq data, regardless of origin (i.e., from species with or without available whole-genome sequences) have mature pipelines (Grabherr et al., 2011; Trapnell et al., 2012). Since, the lines targeted for RNA-seq are not always the same as those used for whole-genome sequencing, these lines may differ from the reference lines due to their diverse breeding histories. To mine our introgression line for candidate genes, we employed combined bioinformatics pipelines for use with and without reference genomes. Using TopHat and Cufflinks software tools, FPKM values and alternative-splicing differences will be uncovered. Using unmapped reads and Trinity software, the possibility of losing important information during transcript assembly is reduced. Therefore, this method is particularly suitable for investigating introgression-like or even hybrid vigor situations with sequenced background genome and exogenous chromosome segment insertions.

Reads not aligned to the reference genome, except for true exogenous transcripts, will crease background noise due to the lack of a complete genome sequence, the presence of gaps or the lack of mitochondrial and chloroplast genomes. As shown in Figure 4, homologous transcripts outside the thickened region should represent false positives. We found that much of the noise can be eliminated by setting the appropriate thresholds for differential gene expression between the trait of interest and the control. However, fold change values often bring another type of noise, especially for genes that are highly expressed in the primary genome. Therefore, experimental validation by PCR or qPCR remains an important step for candidate gene screening. Along with annotations, these filtered candidate transcripts provide much data for further experimental analyses. Due to the rapid increase in whole-genome sequencing projects, our bioinformatics strategy may be applied to similar situations, making mining for candidate genes for interesting traits during introgression breeding more efficient.

## Conclusions

We uncovered gene flow patterns during interspecific hybridization by performing transcriptome analysis of an introgression plant line. Anthocyanin overaccumulation-related genes in *B. rapa* introgression line were found in the original *B. rapa* A genome or were transferred from the *Brassica* B genome of *B. juncea*. Among these genes, the *B. juncea* anthocyanin regulatory gene *R2R3-MYB* was the major contributor to the purple leaf phenotype. DNA-level changes provided by donor chromosome segments, such as cold and light response factors, may also influence the leaf color phenotypes of the hybrids through upstream pathways of the anthocyanin regulatory complex. In addition, we developed a powerful pipeline for distinguishing transcripts originating from two genomes sharing a very close relationship and identified some interesting candidate genes.

## Author contributions

RS and ShuZ designed the study. LX conducted the experiments, analyzed the data and drafted the manuscript. FL prepared samples. WQ collected samples. ShiZ, HZ, and PL helped conduct experiments.

## Funding

This research was supported by China Agricultural Research System (CARS-25-A-01), a Chinese 973 Program Grant (2012CB113900) and a Chinese 863 Program Grant (2012AA100100), both to RS. This study was carried out in the Key Laboratory of Biology and Genetic Improvement of Horticultural Crops, Ministry of Agriculture, P.R. China.

### Conflict of interest statement

The authors declare that the research was conducted in the absence of any commercial or financial relationships that could be construed as a potential conflict of interest.

## Supplementary material

Supplementary Table 1 **DEGs of Alignment I**.

Supplementary Table 2 **DEGs of Alignment II**.

Supplementary Table 3 **FPKM values of anthocyanin biosynthesis genes in Alignment I and II**.

Supplementary Table 4 **Mapping statistics of Alignment I and II**.

Supplementary Table 5 **Primers used in PCR and qPCR validations**.
